# Sophisticated Clean Air Strategies Required to Mitigate Against Particulate Organic Pollution

**DOI:** 10.1038/srep44737

**Published:** 2017-03-17

**Authors:** T. Grigas, J. Ovadnevaite, D. Ceburnis, E. Moran, F. M. McGovern, S. G. Jennings, C. O’Dowd

**Affiliations:** 1 School of Physics and Centre for Climate and Air Pollution Studies, Ryan Institute, National University of Ireland Galway, Galway, Ireland; 2 Met Éireann Glasnevin, Dublin 9, D09 Y921, Ireland; 3 Environmental Protection Agency, Clonskeagh, Dublin 14, D14 YR62, Ireland.

## Abstract

Since the 1980’s, measures mitigating the impact of transboundary air pollution have been implemented successfully as evidenced in the 1980–2014 record of atmospheric sulphur pollution over the NE-Atlantic, a key region for monitoring background northern-hemisphere pollution levels. The record reveals a 72–79% reduction in annual-average airborne sulphur pollution (SO_4_ and SO_2_, respectively) over the 35-year period. The NE-Atlantic, as observed from the Mace Head research station on the Irish coast, can be considered clean for 64% of the time during which sulphate dominates PM_1_ levels, contributing 42% of the mass, and for the remainder of the time, under polluted conditions, a carbonaceous (organic matter and Black Carbon) aerosol prevails, contributing 60% to 90% of the PM_1_ mass and exhibiting a trend whereby its contribution increases with increasing pollution levels. The carbonaceous aerosol is known to be diverse in source and nature and requires sophisticated air pollution policies underpinned by sophisticated characterisation and source apportionment capabilities to inform selective emissions-reduction strategies. Inauspiciously, however, this carbonaceous concoction is not measured in regulatory Air Quality networks.

Atmospheric aerosols from natural and anthropogenic sources are responsible for a range of striking visual and aesthetic features of our skylines, including stunning sunsets, morning haze, blue haze over forests, and complex cloud formations. These features arise from their direct interaction with solar radiation and their roles in cloud formation, where they serve as condensation sites for water vapour. As a consequence, aerosols have both direct and indirect (through influencing cloud formation) effects on the global radiation budget^1^. This is predominantly a cooling effect which acts to mask, at least temporarily, warming by greenhouse gases. However, while atmospheric aerosols are typically invisible, they constitute a critical and ubiquitous component of the air we breathe and can represent a considerable threat to human health, contributing to respiratory and cardiovascular problems, leading to reduced lifespan^2^,^3^. This is exemplified by the severe local air pollution events such as the London smog^4^ events of the 1950’s, leading to the Clean Air Act in 1956. Even the Dublin smog of the 1980s led to significant ‘excess’ mortality rates^5^, resulting in a ban on smoky coal to reduce air pollution. The occurrence of such severe events has largely been reduced in the developed world, although still not eradicated from megacities such as London or Paris and are relatively common in developing megacities such as Beijing, for example.

In addition to the severe smog events, there is increased recognition of the health impacts attributable to exposure to even moderate levels of air pollution. The 2015 EEA report^6^ on Air Quality in Europe reported that 10–14% of the population in Europe was exposed to air pollution concentrations above the EU reference levels for PM_2.5_ and when exposure to the stricter World Health Organisation (WHO) Air Quality Guidelines (AQG) was considered, 91–93% of the population were exposed. The WHO concluded that PM_2.5_ concentrations in 2012 were responsible for about 432,000 premature deaths originating from long-term exposure in Europe^6^. These are associated with photochemically-produced smog^7^ comprising hydrocarbons, nitrogen oxides and ozone and their chemical reaction by-products, including aerosols or PM.

These harmful particulates are classified in terms of the amount of particulate matter smaller than a certain aerodynamic size (50% transmission efficiency): PM_10_ being all the particulate matter mass with aerodynamic diameters smaller than 10 μm. Ambient air quality standards for particulate matter were initially based on measurements of inhalable mass at sizes less than 10 μm^8^, where this size threshold was chosen to limit availability of particles small enough to penetrate beyond the upper airways in the respiratory system^9^. Subsequently, epidemiological studies demonstrated that specifically, fine particle (PM_2.5_) exposure, rather than course particle (PM_2.5–10_), was responsible for the observed association with increased daily mortality rates^2^,^3^ and suggested specific links to combustion related particles. Subsequent work suggested that even smaller, ultrafine, particles with diameters less than 100 nm, could be more important in terms of mortality^10^ and health impacts^11^. These studies, along with a more recent study of magnetite pollution nanoparticles in the human brain^12^, suggested that not only is the mass of ultrafine particle important, but also the source, chemical composition, and number concentration. These studies point to submicron combustion derived particles along with secondary particulate formation as being central to adverse health impacts. However, regulatory metrics are largely based on mass measurement of PM_10_ and PM_2.5_ which can readily be dominated by the contribution of super-micron particles.

In the 1970s, the environmental impacts of air pollution, particularly in the form of acid rain, became a major environmental concern across Europe^13^. This was initially focused on the detrimental regional impacts of sulphur emissions from power generation. Since its establishment in 1979, the UNECE Convention on Long-range Transboundary Air Pollution (CLRTAP) has aimed to address the impacts of a range of air pollutants on human-health, fresh water, forests and other sensitive ecosystems. The 51 Parties to the Convention have established shared systems for pollutant emissions inventories, as well as a range of modelling and analysis programmes; both Canada and the United States have ratified the Convention. In Europe, pollutant monitoring for the Convention is carried out under the European Monitoring and Evaluation Programme (EMEP). Policy actions under CLTRAP aim to reduce emissions of key air pollutants including SO_x_, nitrogen oxides (NO_x_), volatile organic compounds (VOCs) and ammonia (NH_3_). At the EU level, reduction targets agreed under the Convention have been set on a legislative basis under the National Emissions Ceiling Directive http://ec.europa.eu/environment/air/pollutants/ceilings.htm. This is a central element of the Clean Air for Europe (CAFE) programme which was launched in 2001. It identified PM and Ozone as key challenges in terms of reducing pollution levels. Revised CAFE legislative proposals tabled in 2013 are under consideration by the European Parliament and Council. Both CLRTAP and CAFE are developing actions to assess inter-continental and hemispheric aspects of air pollution.

Observations in ‘background’ locations are essential to quantify the extent, influences and impacts of pollutants at regional, hemispheric and global scales as well as in assessing the effectiveness of policy measures. The WMO Global Atmosphere Watch (GAW) programme comprises a global-scale network of such observing stations that are typically more remote than EMEP stations and having a focus on observing essential climate variables^14^. The NE Atlantic is impacted by emissions from Europe and North America and GAW/EMEP stations (Valentia Observatory for the full period since 1980 up to 2014 and Mace Head from 2001 to 2014) is shown in Fig. 1. Here, we present *S* mass concentrations from both non-sea-salt sulphate aerosol (nssSO_4_) mass in the PM_10_ category and from gaseous SO_2_ mass (see Supplementary Information for measurement methods). NssSO_4_ and SO_2_ masses are thrice and twice that of *S*, respectively.

Two questions motivate this study and are subsequently addressed (1) to what extent have air pollution levels reduced over the NE Atlantic since the 1980s? And (2) how clean is current NE Atlantic air?

## Results

### To what extent have air pollution levels reduced over the NE Atlantic since the 1980s?

**Figure 1:**
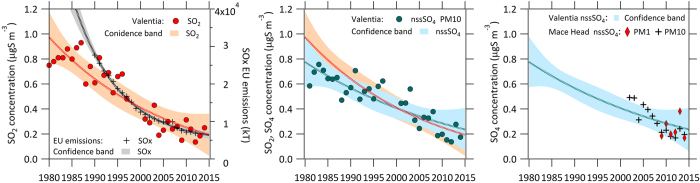
Sulphur air pollution trends. (Left) SO_2_ pollution levels in terms of μg *S* m^−3^ measured at Valentia Observatory over the period of 1980–2014 and European SOx emissions over the period of 1988–2014. Shaded areas represent the confidence bands (95%) of exponential fits. (Middle) SO_2_ confidence bands, as in left panel, overlaid with nss-SO_4_ PM_10_ pollution levels in terms of μg *S* m^−3^ measured at Valentia Observatory over the same time period of 1980–2014 and its confidence bands. (Right) nss-SO_4_ PM_10_, for 2001–2014, and nss-SO_4_ PM_**1**_, for 2009–2014, (again both in terms of μg *S* m^−3^) observed at Mace Head with the 95% confidence bands from Valentia Observatory superimposed.

The 35-year SO_2_ and nss-SO_4_ PM_10_ dataset from Valentia Observatory is central to addressing this question. Figure 1 (left panel) displays the annual average time series for SO_2_ concentrations (for Valentia Observatory), along with the emission inventory for SO_x_ over Europe for the same period. While inter-annual meteorological variability is a prominent feature of the dataset, an overall downward trend in *S* mass concentration, concomitant with a downward trend in SO_x_ emissions, is evident. An exponential trend curve fitted to the data illustrates than the SO_2_ concentration fell from 0.95 μg *S* m^−3^ to 0.2 μg *S* m^−3^, corresponding to a 79% reduction in *S* over the period. The 95% confidence bands of the fit are encapsulated by the shaded area. The centre panel illustrates the non-sea-salt (nss) sulphate (PM_10_) measured at Valentia over the same period. It should be noted that up until 2001, the reported sulphate at Valentia Observatory is total sulphate which includes both secondary sulphate that is formed from the gas phase (e.g. through oxidation of SO_2_) and primary sulphate associated with nascent sea-spay production from bubble bursting and white-capping. The contribution of primary sea-salt sulphate is retrospectively calculated and accounted for in this work from 1980–2001 (see Supplementary Information). The resulting data reveal a reduction of non-sea-salt sulphur from 0.78 μg *S* m^−3^ to 0.22 μg *S* m^−3^ over the period, corresponding to a 72% reduction in *S*. The plate on the right of Fig. 1 illustrates the PM_10_ nss-sulphate from 2001 to 2014 at Mace Head and six years of PM_1_ nss-sulphate mass, also at Mace Head, from 2009–2014. The 95% confidence bands for the complete nss-sulphate record from Valentia Observatory are also displayed for ease of comparison. The Mace Head data exhibit a decreasing trend in PM mass from 2001 up to 2009. The mass concentration data over the 6-year period from 2009–2014 do not exhibit any clear trend, and are perhaps indicative of relatively stable mass concentration regime. PM_1_ data for this 6-year period are provided from a unique continuous aerosol mass spectrometry (AMS) measurement dataset comprising analysis of key pollutant species in addition to nss-sulphate (e.g. nitrate, ammonium, anthropogenic organics) as well as other natural species (sea-salt, methane sulphonic acid and biogenic organics). These data are used in a detailed statistical chemical composition analysis presented in the following section for this period. As outlined earlier, while PM_1_ is not a regulatory metric, it encompasses key pollutants arising from combustion and secondary pollutants that are increasingly being linked to adverse health impacts.

Overall, the 35-year observational record illustrates a reduction of 79% in SO_2_ levels and a 72% in nss-sulphate levels over this period, which strongly tracks the SO_x_ anthropogenic emissions trend. The data include all air mass sectors and types and, therefore, both natural and anthropogenic nss-*S* contributions are included in the trend; however, *S*-isotopic source apportionment studies previously undertaken in the location^15^,^16^ found up to 30% natural *S* contributions in summer months, falling to negligible levels in winter months, leading to an estimated annual contribution of the order of 5–10%.

### How clean is current NE Atlantic air?

The current-day chemical composition of PM_1_ at Mace Head is used to address this question. In fact, as a secondary objective, which relates to the amount of time, if any, that the background location of Mace Head can be regarded as effectively representative of the natural background marine environment is also addressed. These questions are answered through the deployment of a high-resolution, online aerosol mass spectrometric (AMS) which is capable of characterising PM_1_ chemical composition for effectively all species except for Black Carbon (BC). BC was measured using the Multi-Angle Absorption Photometer (*MAAP*). Sampling was performed in all-sector air arriving at Mace Head continuously from 2009–2014. We use BC mass concentration (which is an excellent tracer for anthropogenic and combustion pollution^17^) to classify air hosting different degrees of pollution (see Supplementary Information for measurement technique). For analytical purposes, five classifications of Northern Hemisphere (NH) mid-latitude air pollution levels are defined: NH pristine air having less than 0.015 μg m^−3^ BC; clean air having BC levels between 0.015 and 0.05 μg m^−3^; moderately polluted air with BC between 0.05 and 0.3 μg m^−3^; polluted air as air having BC between 0.3 and 1 μg m^−3^; and lastly, extremely-polluted air as air with BC greater than 1 μg m^−3^. See Supplementary Information for further detail on the classification. It should be noted that the NH pristine air classification, based on a measurable amount of BC, is considered to reflect the ubiquitous nature of anthropogenic influences on the atmosphere of the Northern Hemisphere. However, we distinguish pristine air from clean air at the given threshold additionally on the basis that no anthropogenic OM is detectable by the AMS at BC mass concentrations less than 0.015 μg m^−3^.

**Figure 2:**
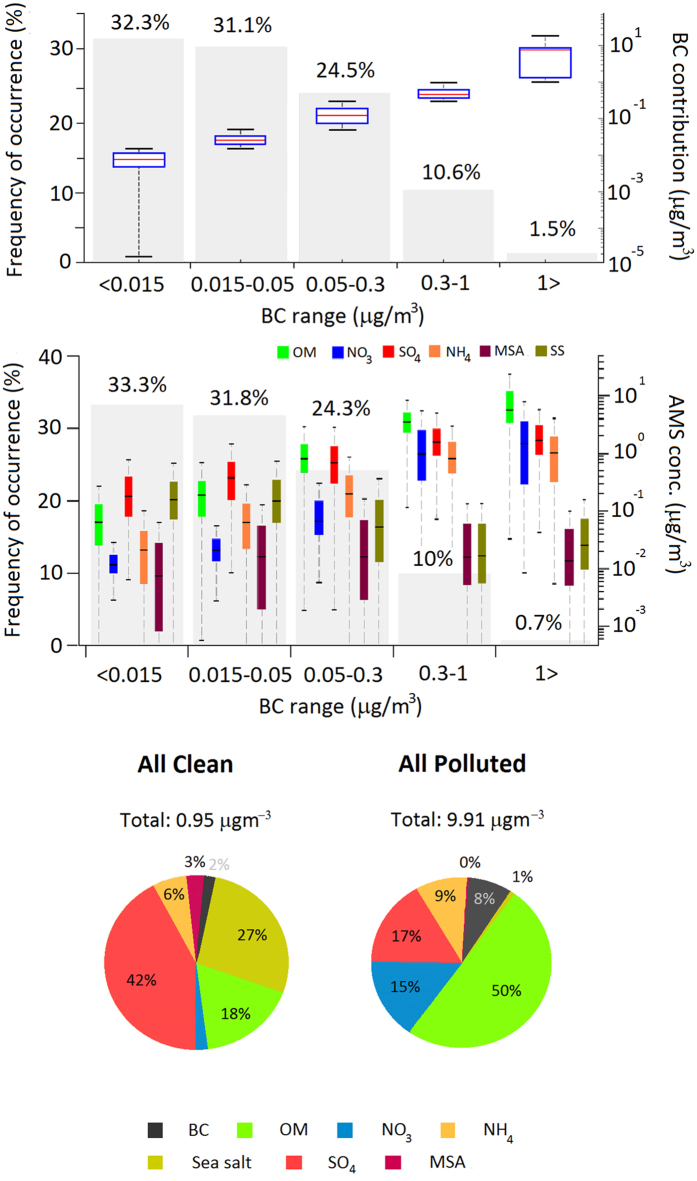
(Top) Box and whisker plots of frequency of occurrence of Black Carbon (*BC*) mass concentration for five categories of air pollution levels classified according to BC levels. Pristine air has *BC* mass less than 0.015 μg m^−3^, clean is 0.015–0.05 μg m^−3^, moderately polluted is 0.05–0.3 μg m^−3^, polluted is 0.3–1 μg m^−3^ and extremely polluted is *BC* > 1 μg m^−3^. (**Middle**) Box and whisker plots of frequency of occurrence of speciated chemical mass concentration for five categories of air pollution levels of frequency of occurrence of organic matter, nitrate, sulphate, ammonium, MSA and sea salt, as measured by the Aerodyne Aerosol Mass Spectrometer with particle sampling over the PM_1_ size range. (**Bottom**) Pie charts of chemical speciated mass concentrations for organic matter, sulphate, BC, nitrate, MSA, sea salt and ammonium for marine air masses (pristine and clean categories combined) and continental air masses that include all polluted categories.

The results of this statistical analysis are shown in Fig. 2 which reveals that pristine conditions occur for 32% of the time, while clean conditions occur for 31%. Moderately polluted and polluted air occurred for 25% and 11%, respectively. Extreme pollution occurred for 1.5% of the time. The mean value of BC increases from 0.008 μg m^−3^ in pristine air to 1.7 μg m^−3^ in the extreme case. Peak values of 8.6 μg m^−3^ are encountered for the 99% percentile. In summary, clean, including pristine, air occurred for 63% of the time, the remainder being classified as (all) polluted. Hereafter, when we refer to clean air, this includes pristine, and polluted refers to moderate to extremely polluted and peak concentration refers to the 99% percentile.

The mass concentration of the main chemical species derived from the AMS dataset is also shown in Fig. 2 and tabulated in detail in Table S1. Sulphate (SO_4_) dominates the chemical mass concentrations in clean air with mean values in the two categories ranging from 0.28 μg m^−3^ to 0.52 μg m^−3^. In polluted air, SO_4_ mean mass ranged from 0.91 μg m^−3^ to 2.1 μg m^−3^ over the three categories. Peak SO_4_ PM_1_ concentrations were 6.4 μg m^−3^.

OM mean mass concentrations ranged from 0.1 μg m^−3^ to 0.24 μg m^−3^ in clean air, and 1.1 μg m^−3^ to 9.8 μg m^−3^ in polluted air. Peak OM concentrations of 79 μg m^−3^ are encountered for biomass burning conditions. Nitrate mean mass concentrations ranged from 0.01 μg m^−3^ to 0.03 μg m^−3^ in clean air and 0.17 μg m^−3^ to 2.6 μg m^−3^ in polluted air, with peak values of 12 μg m^−3^. Ammonium had mean concentration ranges of 0.03 μg m^−3^ to 0.09 μg m^−3^ in clean air and 0.28 to 1.4 μg m^−3^ in polluted air. Peak concentrations were 4.7 μg m^−3^. In terms of relative contributions, in clean air, sulphate mass dominates PM_1_ with a 42% contribution, followed by OM with an 18% contribution. In polluted air, OM prevails with a 50% contribution followed by 17% from sulphate and 15% from NO_3_. Consolidating BC and OM mass into carbonaceous aerosol, this category contributes 60% to polluted PM_1_, and increasing with overall pollution levels to 90% for the most polluted situations.

## Discussion

There are a number of striking outcomes from this analysis: firstly, background NE Atlantic sulfur based air pollution levels have dropped by 72–75% from the peak values measured in the 1980’s. This shows the success of effective policy implemented through regulation and resultant stimulation of technological advances. As a consequence of this reduction, it is evident that for the polluted air categories, OM mass concentrations now make up 50% or more of the PM_1_ mass while sulphate and nitrate contribute to the order of 15–17% each. The total OM mass is typically larger than the sum of both nitrate and sulphate mass, the two dominant inorganic pollutant species, and in extremely polluted conditions OM can constitute up to 90% of the PM_1_ mass.

Secondly, whilst BC is rightly regarded as an important primary aerosol, produced directly by combustion, its contribution to overall PM_1_ mass is relatively minor (<10%) at these remote locations. This compares to 17% in Dublin for PM_1_ (unpublished data) and 5–7% in London for PM_2.5_^18^. Although we don’t have a percentage contribution specifically to PM_1_ in London, the pattern suggests a reduction in the contribution of BC to PM_1_ as the aerosol ages during transport away from its source. This is attributed largely to an increase in the contribution of secondary aerosol as the pollution plumes age during which reactive gaseous species are converted to inorganic PM.

In summary, the average total carbonaceous aerosol makes up 20% of the PM_1_ mass in clean air. It makes up 60% in polluted air. BC and OM levels are well correlated (*r*^2^ = 0.99) over the air mass categories defined (Figure S3), suggesting similar sources for both these aerosol constituents. An OM to BC ratio of 5.7 indicates an aged oxidised aerosol. Finally, there is a strong correlation (*r*^2^ = 0.99) between NH_4_ and the sum of SO_4_ and NO_3_ levels across all the categories defined. This confirms the well-known role of ammonia in production of secondary inorganic aerosol.

Our results are corroborated by recent, shorter term, measurements across Europe^19^ where the dominating contribution to PM_1_ mass by OM is seen in most regions. The highest contributions observed are over Northern Europe, while the highest sulphate contributions are found in Southern Europe with nitrate dominating PM_1_ in certain localised regions (e.g. the Netherlands and at times, in the Po Valley). In summary, for the greater European region, OM is the dominant contributor to PM_1_ air pollution, with its contribution increasing as the degree of pollution increases. A similar picture can also be seen globally^20^.

### Implications for policy

The declining trends in gaseous and particulate *S* concentrations clearly show the success of effective policy aimed at reducing this pollutant, leading to decreased acidification of ecosystems across Europe. However, while sulphate has relinquished its position as the dominant air pollutant species it has revealed new and perhaps more complex challenges for policy if clean air goals are to be achieved. It is well known that for a variety of reasons reductions of NO_x_ and primary PM emissions have not been as successful as anticipated. Ground level ozone levels also remain problematic. The analysis presented here adds a new dimension. It highlights the important contribution of organics to PM mass and strongly suggests that policy intervention aimed at reducing regional PM levels needs to address emissions of organic species.

Current policy does address emissions of Volatile Organic Compounds (VOCs) that are considered to be linked to ground level ozone production rather than PM. As a consequence, a limited set of VOCs either are included in pollutant emissions inventories or have emissions reductions targets. If the contribution of organic species to ambient PM levels is to be addressed then there is a need to further assess the sources and contributions of these emissions, and those of other potential precursor organic compounds such as fatty acids^21^ to ambient PM levels. This requires increased understanding of emission sources and their atmospheric evolution which can be achieved through the linked development of emissions inventories and sophisticated observation and monitoring systems which, in combination, can better inform policy interventions and better assess their effectiveness. The new challenge is to address the complex concoction of widely diverse organic compounds, arising from a diverse range of anthropogenic emissions and to inform and implement policies to address these emissions. This challenge is not insignificant; it is well established that atmospheric organic aerosol exists as a complex mixture of thousands of individual organic compounds^22^,^23^,^24^,^25^,^26^,^27^,^28^.

In this context, if the EU reference air quality standards aim at long term convergence with the WHO AQGs we recommend that a process be established to improve source apportionment knowledge and capabilities through the deployment of advanced next-generation operational measurement systems, such as operational aerosol mass spectrometers in strategic nodes in existing regulatory Air Quality networks while providing local to continental scale analysis. There is a parallel need to ensure that emissions of primary and precursor species are captured in official inventories. In combination, these systems can both inform and measure the effectiveness of appropriate measurable policy responses.

## Additional Information

**How to cite this article:** Grigas, T. *et al*. Sophisticated Clean Air Strategies Required to Mitigate Against Particulate Organic Pollution. *Sci. Rep.* **7**, 44737; doi: 10.1038/srep44737 (2017).

**Publisher's note:** Springer Nature remains neutral with regard to jurisdictional claims in published maps and institutional affiliations.

## Supplementary Material

Supplementary Information
