# Supplementary material for: Sophisticated Clean Air Strategies Required to Mitigate Against Particulate Organic Pollution
Source: Sci Rep. 2017 Mar 17;7:44737. doi: 10.1038/srep44737 (PMC5356191; doi:10.1038/srep44737)
Supplement: Supplementary Information [file srep44737-s1.pdf]

Supplementary Information

Sophisticated Clean Air Strategies Required to Mitigate Against Particulate Organic Pollution

Grigas[1], T., J. Ovadnevaite[1], D. Ceburnis[1], E. Moran[2], F.M. McGovern[3], S.G. Jennings[1], and C. O'Dowd[1].

[1]School of Physics and Centre for Climate and Air Pollution Studies, Ryan Institute, National University of Ireland Galway, Galway, Ireland, [2]Met Éireann, Glasnevin, Dublin 6, [3]Environmental Protection Agency, Dublin.

Correspondence to: (colin.odowd@nuigalway.ie). TG and CO'D are joint lead authors.

Supplementary Information

Global Atmosphere Watch (GAW)/European Monitoring and Evaluation Programme (EMEP) observation facilities. The Mace Head Atmospheric Research Station is located in Connemara, County Galway on the Atlantic Ocean coastline of Ireland at 53° 19′ 36′′ N, 9° 54′ 14′′ W and offers a clean sector from 190° through to 300°. Meteorological records show that, on average, over 60% of the air masses arriving at the station are from the clean sector[1-3]. Air is sampled at 10m height from a main air inlet positioned at 80–120m from coastline depending on tide height (available at: http://www.macehead.org). Valentia Observatory is part of Met Éireann, the Irish Meteorological Service. It is located one kilometre west of the town of Cahirciveen, on the estuary of the Feartha river approximately 5-7 km from the open ocean. The Observatory carries out surface weather and upper-air meteorological measurements, as well as a wide range of other scientific activities including ozone monitoring, geomagnetics, seismology, solar radiation and environmental monitoring. Its coordinates are 51° 56' 23"N - 10° 14'40"W. (http://www.met.ie/about/valentiaobservatory/)

Aerosol measurements. Off-line 24 hour PM_{10} filter samples for sulphate and sulphur dioxide analysis were collected at Valentia since 1980. Particulate matter samples for the analysis of inorganic ions including sulphate were collected with PM_{10} size selective inlet at 2m above ground level. Particulate matter and sulphur dioxide samples were collected using sequentially mounted untreated and impregnated Whatman 40 filters - a 2-stage filter pack method approved at EMEP sites[4]. The measurement programme at Mace Head started in 2001 on a 10 m tower using PM_{10} size selective inlet and PTFE filters for inorganic ion analysis only. Valentia samples were analysed by the Thorin method from 1980 until 1992 and by ion chromatography since 1992. Mace Head samples were analysed only using ion chromatography for a set of inorganic ions including sulphate. Regular filter blanks were collected at both locations. Sample frequency was daily at both locations and yearly average concentrations were produced by taking arithmetic average of daily values.

Sulphate concentrations at Valentia included sea salt sulphate until 2003 after which time a proper correction was enabled using Na data. Sea salt sulphate contribution was subtracted from the total sulphate as 0.25 x Na and presented as non-sea-salt (nss) sulphate from 2004 onwards. Total sulphate yearly average concentrations at Valentia Observatory prior to 2004 were corrected using yearly median concentrations of sea salt sulphate after 2004 as is presented in Figure 1. Figure S2 demonstrates that the correction for the sea salt sulphate contribution prior to 2004 was between 10-20%. Mace Head samples were corrected for sea salt sulphate contribution since the start of the

measurements in 2001. Sampling of PM-sulphate *via* filter collection can be subject to positive artefacts of the order of 8-11%[5].

On-line Aerosol chemical composition was measured with the Aerodyne Research Inc. high resolution time-of-flight Aerosol Mass Spectrometer (*AMS*) which provides real-time size resolved composition analysis of volatile and semi-volatile particulate matter[6,7]. The combination of size and chemical analysis of PM_1 aerosol mass loading with fast time resolution makes the *AMS* unique. The *AMS* was regularly calibrated with 300 nm ammonium nitrate particles, baseline levels of checked weekly as well as cleaning of the critical orifice was performed as required. In summary, the *AMS* quantifies non-refractory aerosol chemical composition, including major inorganic species such as ammonium, sulphate, nitrate, plus organic matter speciation which enables source apportionment. In addition, recent work has demonstrated that both sea salt mass and primary marine organic mass can be retrieved from the high resolution *AMS*[8]. Therefore, AMS sulphate data were also corrected for sea salt contribution which was negligible (~7% of submicron sea salt content). The original AMS time resolution of 5 min was first reduced to daily average concentration and then averaged for a year to compare with filter measurements. For classification purposes AMS time resolution of 5 min was reduced to hourly average concentrations.

Black carbon measurements were performed with the Thermo scientific (Thermo Fisher Scientific Inc., Waltham, MA) multi-angle absorption photometer (MAAP) instrument, model 5012. This instrument calculates absorbance from particles deposited on a filter using measurements of both transmittance and reflectance at two different angles[9]. The mass accommodation coefficient value used was 6.6 m^2 g^{-1} at 670 nm and the measurements were performed at a flow rate of 10 litres min^{-1}. Black carbon is mostly confined to submicron size range, but PM_1 size selective inlet was installed to limit sampling of mineral dust or any other absorbing material in the supermicron size range. The original BC time resolution of 5 min was reduced to hourly average concentrations for further analysis.

Classification of Pollution Categories (Pristine, Clean, Moderately Polluted, Polluted and Extremely Polluted): **Classification of pollution categories was done using hourly average concentrations of species which were obtained from native 5 min resolution of BC and AMS data.** Based on the BC frequency distribution (Figure S3), five black carbon ranges were selected for the statistical analysis of the aerosol properties in North-East Atlantic. Lognormal distributions were assumed to result from dilution of BC concentrations while moving away from its source. Four modes were found to represent the BC frequency distribution at Mace Head for the period of interest. Therefore, the classification of pollution categories, or threshold BC values separating the categories, were defined on the basis each pollution category encapsulating values that lie within two standard deviations (2σ, or 95.45%) of the lognormal distribution mode. Four modes resulted in four pollution ranges plus additional one to include all values above 1 μg m^{-3}. Ranges were named accordingly: I) Pristine with BC threshold of 0.015 μg m^{-3}; II) Clean air as having BC between 0.015 and 0.05 μg m^{-3}; III) Moderately polluted is classified as air with BC between 0.05 and 0.3 μg m^{-3} while IV) Polluted is classified as 0.3 to 1 μg m^{-3} and finally, V) extremely polluted as greater than 1 μg m^{-3}. Although the first mode resulted in the limit of 0.018 μg m^{-3}, 0.015 μg m^{-3} limit was selected to be consistent with previous studies[10] that showed trace evidence of refined hydrocarbons in the AMS mass spectra, and thus, some anthropogenic contribution to the OM, for BC larger than 0.015 μg m^{-3}. Clearly, however, if one is using BC as a tracer, and it is always positive to some degree, there can be no place that is truly 100% pristine.

The three remaining upper thresholds were rounded up or down to the nearest convenient number: e.g. 0.046 μg m^{-3} to 0.05 μg m^{-3}, 0.36 μg m^{-3} to 0.3, μg m^{-3} and 0.95 μg m^{-3} to 1 μg m^{-3}. These four

ranges are consistent with different dilution extents: the largest concentration mode, Mode 4, can be atributed to local sources, Mode 3 to European outflow diluted ~4 times and Mode 2 to North American long-range transport diluted a further 4-5 times. The dilution variation was consistent with the distances from continental Europe (1,500km) and North America (3,000km) to measurement locations.

References SI

1 O'Dowd, C. *et al.* Do anthropogenic, continental or coastal aerosol sources impact on a marine aerosol signature at Mace Head? *Atmos Chem Phys* **14**, 10687-10704, *10.5194/acp-14-10687-2014* (2014).

2 Jennings, S. G. *et al.* Mace head atmospheric research station characterization of aerosol radiative parameters. *Boreal Environ Res* **8**, 303-314, (2003).

3 O'Connor, T. C., Jennings, S. G. & O'Dowd, C. D. Highlights of fifty years of atmospheric aerosol research at Mace Head. *Atmos Res* **90**, 338-355, *DOI:10.1016/j.atmosres.2008.08.014,* (2008).

4 Bashir, W. *et al.* Chemical trends in background air quality and the ionic composition of precipitation for the period 1980-2004 from samples collected at Valentia Observatory, Co. Kerry, Ireland. *J Environ Monitor* **10**, 730-738, *DOI:10.1039/B803010C* (2008).

5 Tsai, Y. I., Sopajaree, K., Kuo, S.C., Hsin, T. Y., Artifacts of ionic species for hi-vol PM_{10} and PM_{10} dichotomous samplers, Atmospheric Environment 32(9):1605–1613, *DOI: 10.1016/S1352-2310(97)00387-7*, (1998).

6 Jimenez, J. L. *et al.* Ambient aerosol sampling using the Aerodyne Aerosol Mass Spectrometer. *J Geophys Res-Atmos* **108**, 8425, *DOI:10.1029/2001JD001213* (2003).

7 DeCarlo, P. F. *et al.* Field-deployable, high-resolution, time-of-flight aerosol mass spectrometer. *Anal Chem* **78**, 8281-8289, *DOI:10.1021/Ac061249n* (2006).

8 Ovadnevaite, J. *et al.* On the effect of wind speed on submicron sea salt mass concentrations and source fluxes. *J Geophys Res-Atmos* **117**, *DOI:10.1029/2011jd017379* (2012).

9 Petzold, A. & Schönlinner, M. Multi-angle absorption photometry—a new method for the measurement of aerosol light absorption and atmospheric black carbon. *J Aerosol Sci* **35**, 421-441, http://dx.doi.org/10.1016/j.jaerosci.2003.09.005 (2004).

10 Ovadnevaite, J. *et al.* Primary marine organic aerosol: A dichotomy of low hygroscopicity and high CCN activity. *Geophys Res Lett* **38**, *DOI:10.1029/2011gl048869* (2011).

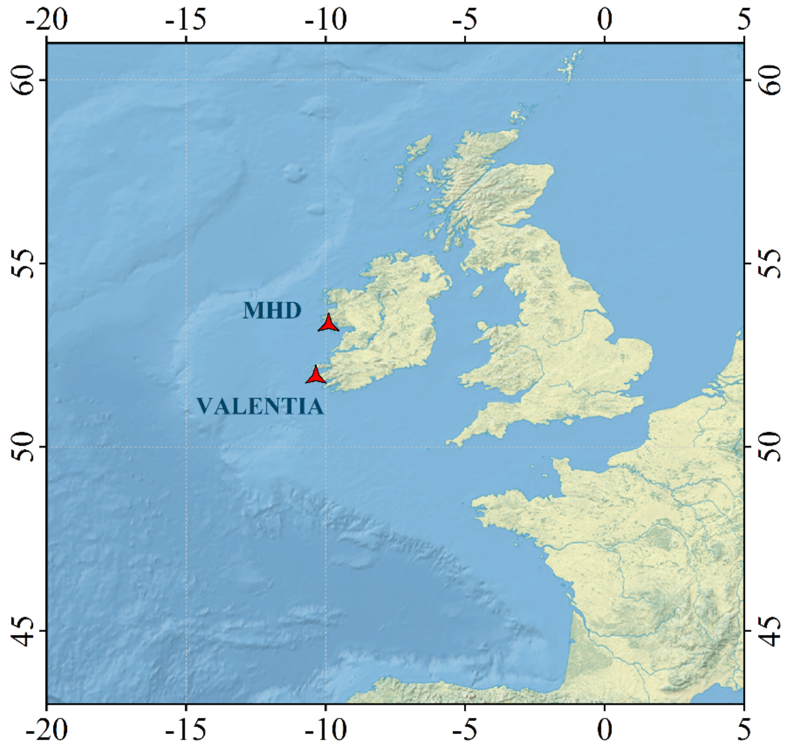

Figure S1: Location of Mace Head and Valentia. The map was created by IGOR Pro 6 (www.wavemetrics.com).

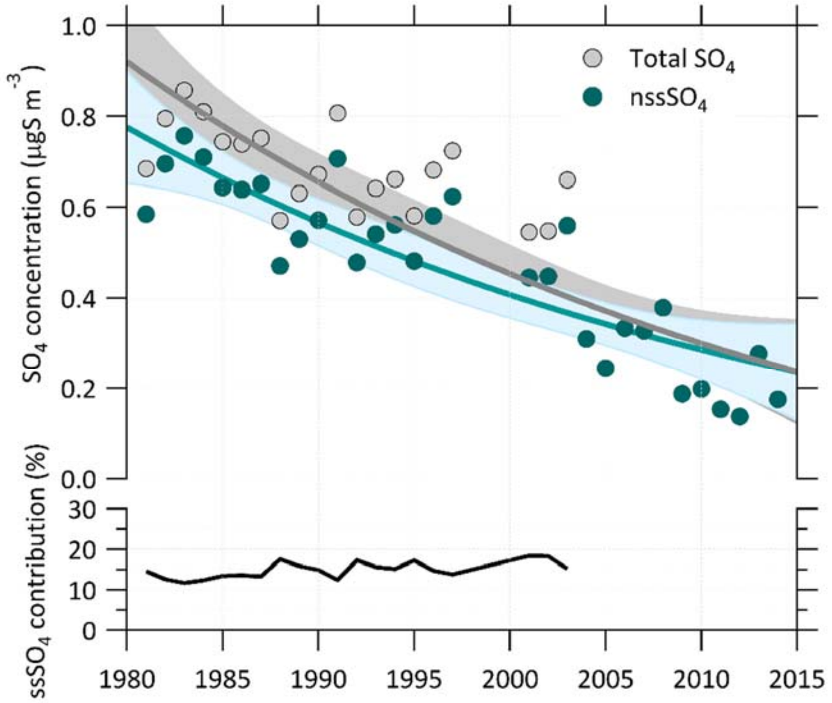

Figure S2. Sulphate concentration trends at Valentia representing total sulphate and corrected nss-sulphate before 2004 and corrected nss-sulphate values after 2004. The trend in grey is derived from a joint time series using total and nss-sulphate data. The light blue trend is derived from corrected nss-sulphate data prior and post 2004. The sea salt sulphate contribution to total sulphate until 2004 is presented in the lower panel and was limited to 10-20%.

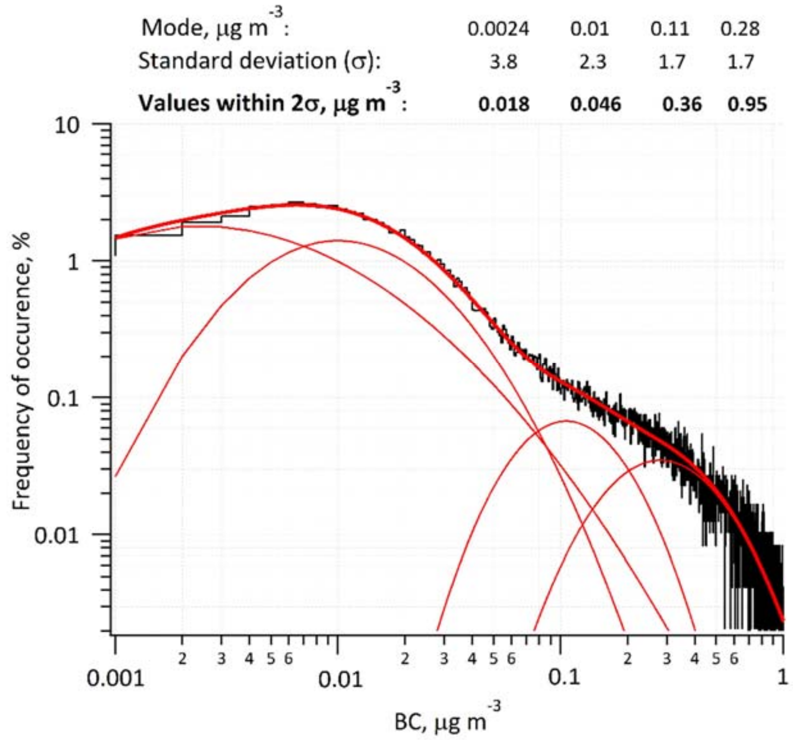

Figure S3 Frequency distribution of the black carbon concentrations at Mace Head for the years of 2009-2015. The distribution was fitted with 4 lognormal modes, where L is the mode location on the x-axis, or in this case, the BC modal value and σ is the standard deviation.

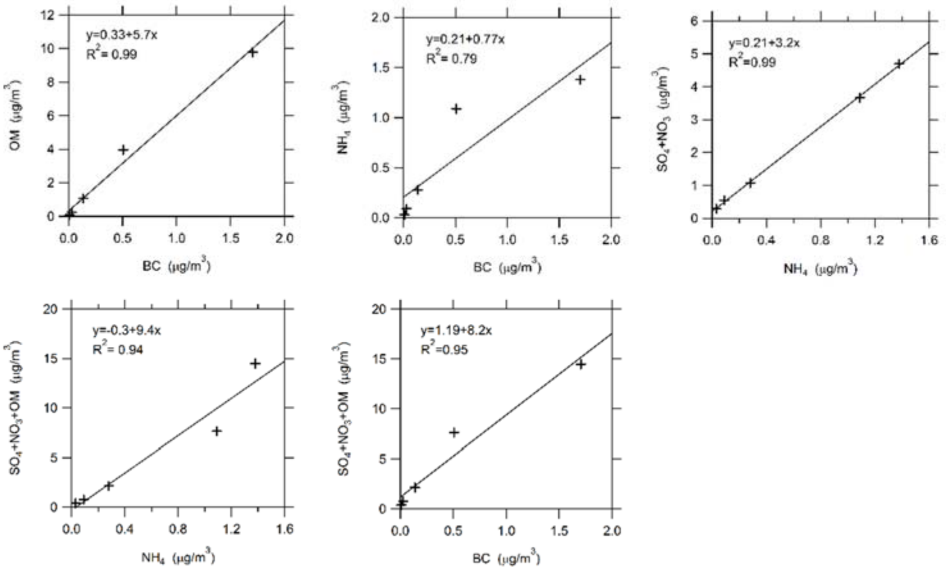

Figure S4. Scatter plots between MAAP-derived BC and AMS-derived PM_1 chemical species mean mass concentrations from 2009-2014.

Table S1. Means, medians and percentiles of aerosol parameters at Mace Head from 2009-2015					
**Parameter**	**Pristine**	**Clean**	**Moderately**	**Polluted**	**Extreme**
**BC μg m^{-3}**	**0-0.015 μg m^{-3}**	**0.015-0.05 μg m^{-3}**	**0.05-0.3 μg m^{-3}**	**0.3-1 μg m^{-3}**	**>1 μg m^{-3}**
99%	0.015	0.049	0.29	0.97	8.6
75%	0.011	0.035	0.19	0.62	1.5
Mean	0.008	0.027	0.14	0.51	1.7
Median	0.007	0.025	0.12	0.47	1.2
25%	0.004	0.019	0.074	0.37	1.1
**Sea Salt μg m^{-3}**	**Pristine**	**Clean**	**Moderately**	**Polluted**	**Extreme**
99%	1.3	1.8	1.2	0.68	0.54
75%	0.32	0.35	0.15	0.06	0.04
Mean	0.25	0.27	0.13	0.06	0.04
Median	0.16	0.15	0.05	0.02	0.02
25%	0.07	0.06	0.01	0.00	0.00
**OM μg m^{-3}**	**Pristine**	**Clean**	**Moderately**	**Polluted**	**Extreme**
99%	0.54	1.1	4.1	12	79
75%	0.13	0.33	1.4	5	12
Mean	0.10	0.24	1.1	4	9.8
Median	0.06	0.19	0.80	3.6	6.3
25%	0.03	0.08	0.46	2.4	3.8
**NO_3 μg m^{-3}**	**Pristine**	**Clean**	**Moderately**	**Polluted**	**Extreme**
99%	0.04	0.09	2	8.8	12
75%	0.02	0.03	0.15	2.5	3.8
Mean	0.01	0.03	0.17	1.7	2.6
Median	0.01	0.02	0.07	0.99	1.9
25%	0.01	0.01	0.04	0.35	0.56
**SO_4 μg m^{-3}**	**Pristine**	**Clean**	**Moderately**	**Polluted**	**Extreme**
99%	1.3	2.3	3.7	6.4	6.4
75%	0.38	0.71	1.3	2.6	3
Mean	0.28	0.52	0.91	1.9	2.1
Median	0.18	0.38	0.70	1.6	1.8
25%	0.08	0.16	0.31	0.97	1.1
**NH_4 μg m^{-3}**	**Pristine**	**Clean**	**Moderately**	**Polluted**	**Extreme**
99%	0.19	0.47	1.4	3.8	4.7
75%	0.04	0.13	0.40	1.5	1.9
Mean	0.03	0.09	0.28	1.1	1.4
Median	0.02	0.06	0.20	0.83	1.1
25%	0.00	0.02	0.08	0.46	0.45
**MSA μg m^{-3}**	**Pristine**	**Clean**	**Moderately**	**Polluted**	**Extreme**
99%	0.17	0.31	0.36	0.31	0.38
75%	0.03	0.05	0.07	0.06	0.04
Mean	0.02	0.04	0.05	0.05	0.04
Median	0.01	0.02	0.02	0.02	0.01
25%	0.00	0.00	0.00	0.01	0.00
